# Are the IL-2 Receptors Expressed in the Murine Fetal Thymus Functional?

**DOI:** 10.1155/1990/59576

**Published:** 1990

**Authors:** Juan Carlos Zoúñiga-Pflücker, Kendall A. Smith, Lucio Tentori, Drew M. Pardoll, Dan L. Longo, Ada M. Kruisbeek

**Affiliations:** 1 Biological Response Modifiers Program National Cancer Institute Bethesda Maryland 20892 USA; 2 Dartmouth Medical School Hanover New Hampshire 03756 Germany; 3 Instituto di Medicina Sperimentale CNR Universita Degli Studi Roma Italy; 4 Johns Hopkins Medical School Baltimore Maryland 21205 USA; 5 BRMP, NCI National Institutes of Health Bldg. 10, Rm. 12N 226 Bethesda Maryland 20892 USA

## Abstract

It is well established that the majority of murine fetal thymocytes (day 15 of gestation) express receptors for interleukin 2 (IL-2), but the functional significance of these IL-2 receptors (IL-2Rs) is not clear. In situ hybridization data show a developmentally regulated expression of IL-2 and IL-2R mRNA. IL-2 binding studies were performed on fetal thymocytes and the results show the presence of both high (kD ≅ 20 pM) and low (kD ≅ 10 nM) affinity IL-2Rs. These IL-2Rs are indeed functional: intact fetal thymic lobes (but not cell suspensions) cultured in IL-2 exhibited an in vitro proliferative response at 20 pM of IL-2, corresponding with the presence of a functional high-affinity IL-2R on fetal thymocytes. The IL-2-dependent growth was primarily observed in the IL-2R + thymic subset, which contains the CD3-/CD4-/CD8- precursor thymocytes. Furthermore, in vitro blocking of IL-2 in intact fetal thymic lobes resulted in a reduction in the cell yield, which predominantly affected the expansion of the immature CD3-/CD4-/CD8-thymocytes. Our findings strongly support the concept that the IL-2/IL-2R pathway is responsible for the proliferation of precursor cells within the fetal thymus.


*Developmental Immunology*, 1990, Vol. 1, pp. 59–66
Reprints available directly from the publisher
Photocopying permitted by license only

© 1990 Harwood Academic Publishers GmbH
Printed in the United Kingdom

### Are the IL-2 Receptors Expressed in the Murine Fetal Thymus Functional?

JUAN CARLOS ZÚÑIGA-PFLÜCKER,*† KENDALL A. SMITH,‡ LUCIO TENTORI,§ DREW M. PARDOLL,¶ DAN L. LONGO† and ADA M. KRUISBEEK†

†*Biological Response Modifiers Program, National Cancer Institute, Bethesda, Maryland 20892*
‡*Dartmouth Medical School, Hanover, New Hampshire 03756*
§*Instituto di Medicina Sperimentale, CNR Universita Degli Studi Roma, Italy*
¶*Johns Hopkins Medical School, Baltimore, Maryland 21205*

It is well established that the majority of murine fetal thymocytes (day 15 of gestation) express receptors for interleukin 2 (IL-2), but the functional significance of these IL-2 receptors (IL-2Rs) is not clear. In situ hybridization data show a developmentally regulated expression of IL-2 and IL-2R mRNA. IL-2 binding studies were performed on fetal thymocytes and the results show the presence of both high (kD ≅ 20 pM) and low (kD ≅ 10 nM) affinity IL-2Rs. These IL-2Rs are indeed functional: intact fetal thymic lobes (but not cell suspensions) cultured in IL-2 exhibited an in vitro proliferative response at 20 pM of IL-2, corresponding with the presence of a functional high-affinity IL-2R on fetal thymocytes. The IL-2-dependent growth was primarily observed in the IL-2R+ thymic subset, which contains the CD3−/CD4−/CD8− precursor thymocytes. Furthermore, in vitro blocking of IL-2 in intact fetal thymic lobes resulted in a reduction in the cell yield, which predominantly affected the expansion of the immature CD3−/CD4−/CD8− thymocytes. Our findings strongly support the concept that the IL-2/IL-2R pathway is responsible for the proliferation of precursor cells within the fetal thymus.

KEYWORDS: IL-2, IL-2 receptor, T-cell development, precursor thymocytes.

### INTRODUCTION

It is now accepted that interleukin 2 (IL-2) is responsible for promoting the growth of mature T cells subsequent to their activation by antigen (Smith, 1984). By comparison, the role of IL-2 in directing the proliferation of immature T cells remains controversial, particularly as proliferation occurs maximally in the developing thymus before expression of the T-cell antigen receptor complex (TCR). By day 15 of murine fetal development, a majority of fetal thymocytes (>60%) express membrane receptors for IL-2, as evaluated by staining with monoclonal antibodies (mAbs) reactive with the IL-2 receptor (IL-2R) (Raulet, 1985; Habu et al., 1985; von Boehmer et al., 1985; Penit and Vasseur, 1989). Fetal thymocytes also have the capability to produce IL-2 when stimulated in vitro (Ceredig, 1986; Tetori et al., 1988a). Therefore, it is plausible to hypothesize that the IL-2/IL-2R pathway is somehow involved in T-cell development, providing growth and/or differentiation signals. However, several reports have questioned the functional status of the IL-2R on fetal thymocytes (Ceredig et al., 1985; Lowenthal et al., 1986), in part because the mAbs used to detect the IL-2R identify only the p55 chain of the IL-2R (Saragovi and Malek, 1987; Teshigawara et al., 1987). This chain binds IL-2 with low affinity and cannot deliver the growth signal to the cell in the absence of the p75 chain; both chains are required to form functional high-affinity IL-2Rs (Smith, 1988).

The first and most crucial question regarding the possible role of the IL-2/IL-2R pathway in T-cell development is whether the IL-2R expressed on fetal thymocytes is indeed functional. Answers to this question so far have been diverse. Although several

*Corresponding author. Present address: BRMP, NCI, National Institutes of Health, Bldg. 10, Rm. 12N 226, Bethesda, Maryland 20892.

studies report that murine fetal thymocytes do not respond to IL-2 by proliferation (Raulet, 1985; Habu et al., 1985; von Boehmer et al., 1985), other studies show that both murine and human fetal thymocytes exhibit IL-2-promoted proliferation (Hardt et al., 1985, 1986; Toribio et al., 1988; Ceredig et al., 1989; Toribio et al., 1989; Watson et al., 1989). The controversy is further fueled by the observation that the affinity of IL-2Rs on resting double-negative (CD4−/CD8−) adult thymocytes (regarded as a model for fetal thymocytes) is lower than that reported for IL-2Rs on mature activated T cells (Ceredig et al., 1985; Lowenthal et al., 1986). Additionally, the IL-2Rs on these adult double-negative thymocytes cannot internalize IL-2. Even more compelling is the lack of any data showing that adequate concentrations of IL-2 are produced in the thymus. Given these considerations, we were intrigued by the observation that mAbs against the IL-2R p55 chain have dramatic inhibitory effects on fetal thymocyte maturation and proliferation (Jenkinson et al., 1987; Tentori et al., 1988b).

In view of all these data, we sought to determine directly whether both IL-2 and functional IL-2R are expressed during T-cell development, and whether fetal thymocytes can proliferate in response to the low concentrations of IL-2 predicted to saturate high-affinity IL-2Rs. Our results indicate that IL-2 and IL-2R mRNA undergo developmentally regulated expression, and that the IL-2Rs expressed in fetal thymocytes are functional high-affinity receptors. The present data also suggest a role for IL-2 during the expansion of precursor thymocytes, thus supporting the concept that the IL-2/IL-2R pathway is involved in thymocyte maturation.

### RESULTS AND DISCUSSION

### Developmental Expression of IL-2 and IL-2R mRNA

We examined the kinetics and population distribution of both IL-2 and IL-2R mRNA expression during fetal ontogeny. Using a quantitative method of in situ hybridization, we demonstrated that IL-2 and IL-2R mRNA are expressed simultaneously in a small fraction of developing murine fetal thymocytes as early as day 13 of gestation (Fig. 1). Moreover, by day 15, the majority of fetal thymocytes express mRNA for IL-2 and IL-2R, and both transcripts decline to low levels by the time of birth. These data provide the first evidence that fetal thymocytes are capable of IL-2 production without exogenous stimulation, and that the entire IL-2/IL-2R system appears to be developmentally regulated (Carding et al., 1989). Furthermore, since IL-2 and IL-2Rs are expressed simultaneously, the data satisfy the minimal requirement for implicating IL-2 during fetal thymic development.

### Analysis of High- and Low-Affinity Receptors Expressed by Fetal Thymocytes

Mature antigen-activated T cells require functional high-affinity IL-2Rs to respond to the growth-promoting signals provided by IL-2 (Smith, 1988). Since previous experiments with fetal thymocytes only examined the expression of nonfunctional, low-affinity, IL-2 binding sites using anti-p55 mAbs (Habu et al., 1985; Raulet, 1985; Penit and Vasseur, 1989), we performed equilibrium binding assays with [125-I]-IL-2 to monitor both high- and low-affinity binding sites. Compared with the IL-2-de-

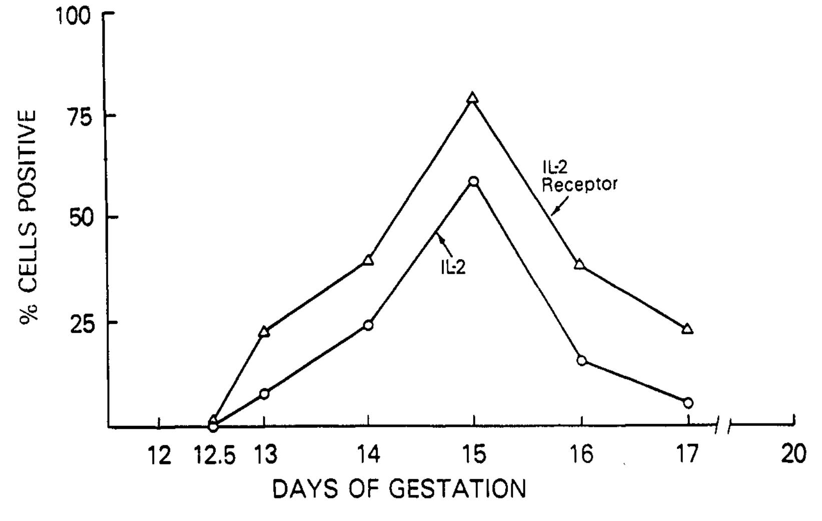

FIGURE 1. In situ hybridization of fetal thymocytes for IL-2 and IL-2R expression: Time course of mRNA expression.

pendent cell line CTLL-2 (Gillis and Smith, 1977) (Fig. 2c), day 15 (Fig. 2a) and day 16 (Fig. 2b) fetal thymocytes express remarkably similar IL-2 binding sites of both high (kD = 7–29 pM) and low (kD = 7–16 nM) affinity receptors. Thus, the present results contrast with those previously obtained in resting adult double-negative thymocytes, in which higher kD values were observed (Ceredig et al., 1985; Lowenthal et al., 1986). Failure to detect high-affinity IL-2 receptors in adult double-negative thymocytes may have been due to the rigorous isolation procedure of cytotoxic depletion used to enrich for double-negative thymocytes; such lengthy methodology may disrupt the stability of the IL-2R on the remaining double-negative population. In the present study, IL-2R affinity was examined immediately following fetal thymus dissection and cell-suspension preparation. Therefore, the expression of high-affinity IL-2Rs by fetal thymocytes suggests that these receptors may in fact provide a functional signal during thymic ontogeny.

### IL-2-Dependent Response by Fetal Thymocytes

Given the demonstration of IL-2 and IL-2R p55 mRNA transcripts in developing thymocytes, and having detected high-affinity IL-2R expression, we next tested for IL-2 responsiveness in vitro. In this regard, it is noteworthy that former reports failed to demonstrate a proliferative response by fetal thymocyte single-cell suspensions to IL-2 (Habu et al., 1985; Raulet, 1985; von Boehmer et al., 1985). Our initial attempts to reevaluate the thymocyte response to IL-2 confirmed these previous findings: a proliferative response was observed solely at doses of IL-2 (≅5 nM) significantly higher than that required to saturate the high-affinity receptors (≅20 pM) (data not shown). However, time-course studies indicated that a greater response to lower doses of IL-2 was observed at very early stages after the disruption of the fetal thymic lobes (data not shown). Therefore, we argued that an intact thymic microenvironment might allow for a more physiological IL-2 response (Papiernik et al., 1987; Denning

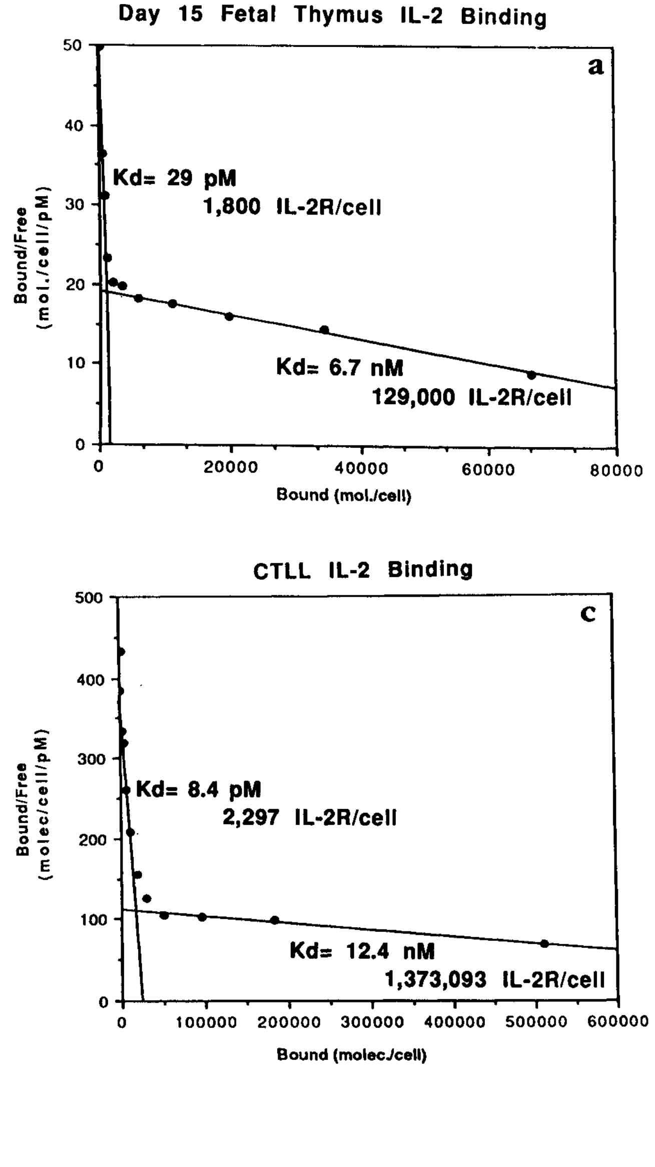

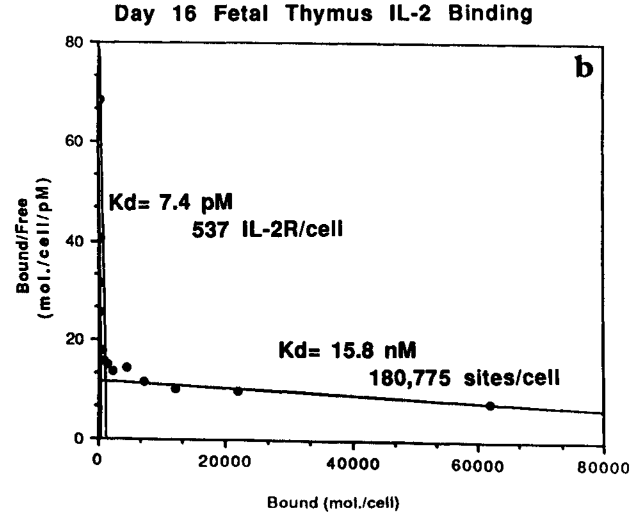

FIGURE 2. IL-2 binding assay of fetal thymocytes. Scatchard plot analysis of [125-I]-rIL-2 equilibrium binding data after substraction of nonspecific binding for freshly isolated (A) day-15 fetal thymocytes, (B) day-16 fetal thymocytes, or (C) the CTLL IL-2-dependent cell line.

et al., 1988). Indeed, Fig. 3 shows that intact fetal thymic lobes cultured in IL-2 exhibit an in vitro proliferative response at 20–40 pM of IL-2, a dose corresponding well with the equilibrium dissociation constant (i.e., kD≅7–29 pM) of high-affinity IL-2Rs. Furthermore, the observed increased proliferation was IL-2 specific as anti-IL-2R (anti-p55) mAbs blocked the response (Fig. 3b). The necessity of an intact thymic microenvironment may reflect the need for cell–cell (thymic stromal cells) interactions (Papiernik et al., 1987; Denning, et al., 1988; Ceredig et al., 1989), which may provide signals and/or other cytokines (IL-7, Watson et al., 1989) necessary for the proper function of the IL-2R.

Next, we examined to what extent the IL-2-induced increase in [3-H]-thymidine incorporation reflected DNA synthesis in IL-2R+ cells. The marked proliferative response to IL-2 by fetal thymic lobes affects primarily those thymocytes that express IL-2Rs, as determined by the increased relative DNA content of IL-2R+ thymocytes (Table 1). In contrast, IL-2R-thymocytes did not exhibit increased relative DNA content.

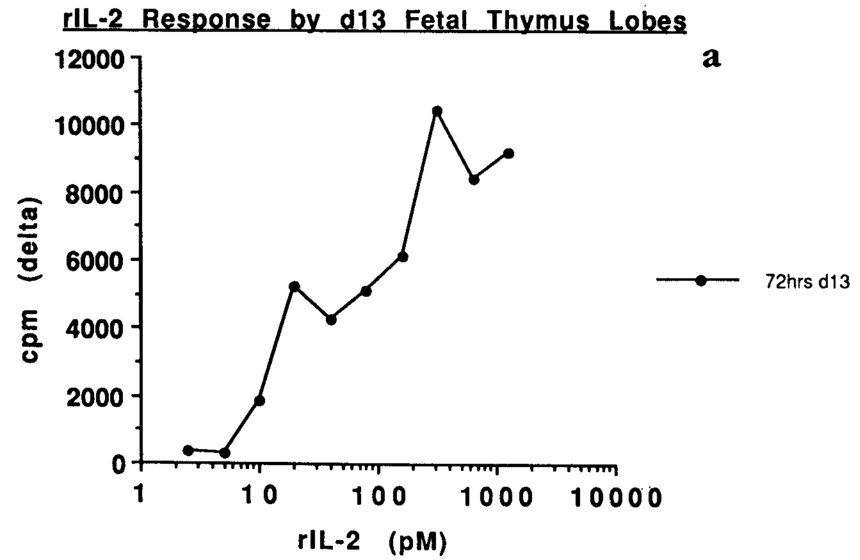

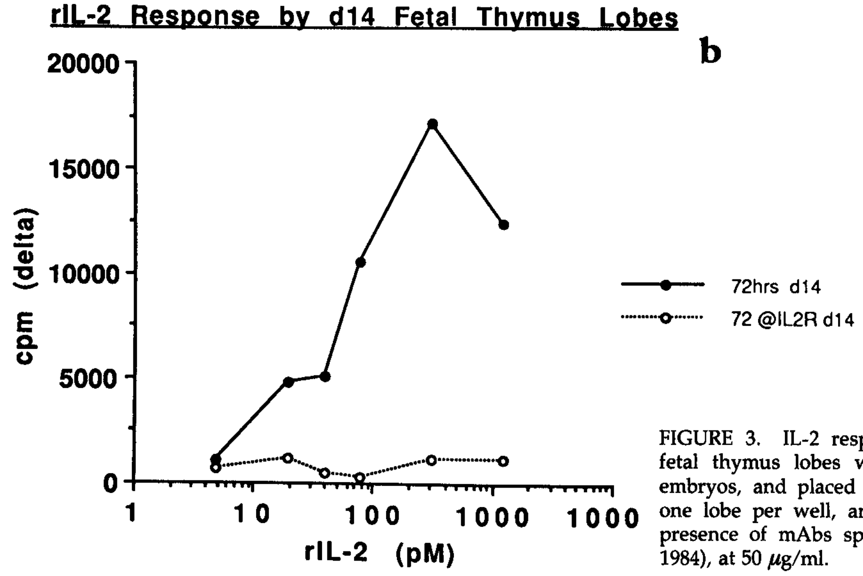

FIGURE 3. IL-2 response by intact fetal thymus lobes. Intact fetal thymus lobes were isolated from (A) day 13 or (B) 14 embryos, and placed in culture in a 96-well microtiter plate, at one lobe per well, and cultured for 72 hr. @IL2R indicates the presence of mAbs specific for the murine IL-2R (Ortega et al., 1984), at 50 μg/ml.

TABLE 1

Effects of rIL-2 Stimulation on Fetal Thymic Lobes[a]

| | % Positive[b] | |
|---|---|---|
| | Control | IL-2 |
| Exp. 1 | | |
| IL-2R+ | 56.5 | 68.5 |
| IL-2R+ high DNA content | 36.7 | 49.2 |
| IL-2R−, high DNA content | 23.0 | 16.5 |
| Exp. 2 | | |
| CD3−/CD4−/CD8− | 25.3 | 48.6 |
| CD3+/CD4−/CD8− | 4.1 | 6.5 |
| Vγ3+/CD4−/CD8− | 3.3 | 7.4 |
| Total CD4−/CD8− | 30.8 | 57.2 |
| CD3+, dull | 35.0 | 27.8 |
| CD3+, bright | 3.6 | 8.2 |
| Exp. 3 | | |
| Total CD4−/CD8− | 31.6 | 55.3 |
| CD3+/CD4−/CD8− | 4.0 | 6.6 |
| IL-2R+ | 42.7 | 48.3 |

[a]Phenotypic analysis of intact day-14 fetal thymic lobes responding to IL-2 (50 pM) in vitro following a 4-day incubation. IL-2R+ cells with high DNA content were Thy-1+, and fetal thymic lobes cultured in IL-2 also showed a marked increase in forward light scatter (data not shown). In both groups, an equivalent number of cells was recovered per lobe. The percent (%) positive indicates positive fluorescence over background, stained with an irrelevant antibody. Results shown are representative of five independent experiments.

[b]Day 14 FTOC, 4-day culture.

Stimulation with IL-2 also increases, most markedly, the absolute and relative number of CD3−/CD4−/CD8− (triple-negative, TN) cells, which doubles following incubation in IL-2 for 96 hr (Table 1). CD4−/CD8− thymocytes are known to include the thymic precursor cells (Fowlkes et al., 1985; Scollay et al., 1988), and their responsiveness to IL-2, reported here, suggests that IL-2 may be involved in the proliferation of these intrathymic multipotent CD3−/CD4−/CD8− T-cell precursors.

In addition, the relative and absolute number's of CD4−/CD8− thymocytes expressing V-gamma-3 TCR (Havran and Allison, 1988) increased after IL-2 stimulation (Table 1), consistent with the recently reported observation that V-gamma-3+ cells in the fetal thymus express high levels of IL-2Rs (Havran and Allison, 1989). An analysis of other V-gamma/V-delta subsets awaits availability of specific staining reagents.

The above data establish the conditions under which IL-2 receptors on fetal thymocytes can deliver growth-promoting signals, and also resolve the controversy regarding the unresponsiveness of IL-2Rs expressed on fetal thymocytes. Furthermore, these data may explain why anti-IL-2R mAbs have such a dramatic blocking effect on T-cell development when applied in situations where the thymic microenvironment is intact (Jenkinson et al., 1987; Tetori et al., 1988b): it is only under the latter conditions that expression of a high-affinity IL-2R translates into the presence of functional IL-2Rs, and that proliferation of several early thymic subsets can be achieved with IL-2. Events such as normal proliferation and differentiation have been shown to occur in vitro only when an intact fetal lobe is placed into culture (Ceredig, 1988; Ceredig et al., 1989), thus emphasizing the importance of an intact thymic microenvironment for the proper cellular interactions and signals to take place. Blocking the IL-2R pathway may delay these early events, thus explaining the anti-IL-2R-mediated alteration in T-cell development (Jenkinson et al., 1987; Tentori et al., 1988b).

### Blockade of the IL-2-Dependent Expansion by Immature Fetal Thymocytes

The previous results suggest that IL-2 may mediate the cell proliferation that occurs within the fetal thymus at a stage when IL-2R expression on TN (CD3−/CD4−/CD8−) thymocytes is maximal (70% by day 15; Penit and Vasseur, 1989), and fetal thymic growth is at an exponential rate (200-fold from day 14 to day 17; Penit and Vasseur, 1989). All these events correlate well with the high expression of IL-2 mRNA and the presence of high-affinity functional IL-2Rs, thus, implicating a role for IL-2 and its receptor during this stage of thymic expansion.

To investigate whether the binding of IL-2 to its receptor on precursor cells is necessary in stimulating cell proliferation, an anti-IL-2 mAb (S4B6; Mossman et al., 1986) was added to intact day-14 fetal thymic organ cultures. Table 2 shows that IL-2 neutralization results in a (≅40%) reduction in cell yield when compared with control cultures of similar duration. Similar results were previously reported for IL-2 receptor blocking in organ culture (Jenkinson et al., 1987). Consistent with our findings that TN thymocytes exhibit an IL-2-dependent expansion, it can be expected that an anti-IL-2 blockade might specifically affect this subset most dramatically. Indeed, Table 3 shows that the relative and absolute number of immature thymocytes (TN, and CD5-dull or Ly1-dull; Fowlkes et al., 1985) is reduced under such conditions. It is noteworthy that the IL-2R expression in the anti-IL-2-treated group is obliterated; this is consistent with earlier findings that show that IL-2 regulates the expression of its own receptor (Malek and Ashwell, 1985). Therefore, the observed effects from the anti-IL-2 blocking may not only reflect an absence of IL-2, but also reflect a down modulation of the IL-2R, resulting in a deficient IL-2/IL-2R pathway. Whatever the mechanism, a reduction in cell proliferation and expansion of the thymocyte precursor pool is observed.

TABLE 2
Cell Recovery Following IL-2 Blocking in Fetal Thymus Organ Cultures[a]

| | Recovery: Cells/Lobe ($\times 10^6$) | |
|---|---|---|
| | Control | Anti-IL-2 |
| Exp. 1[b] | 0.54 | 0.29 |
| Exp. 2[b] | 0.34 | 0.23 |
| Exp. 3[c] | 0.10 | 0.04 |
| Exp. 4[c] | 0.15 | 0.10 |

[a]Cellularity and viability of the intact fetal thymic organ cultures were determined by trypan blue exclusion. Cell recovery corresponds to thymic lobes pooled from each group (usually 36 lobes/treatment group). Anti-IL-2 (S4B6; Mossman et al., 1986) treatment: S4B6 mAb was added daily at a final concentration of 50 μg/ml.
[b]Day 14 FTOC, 9-day culture.
[c]Day 14 FTOC, 6-day culture.

TABLE 3
Effects of Anti-Il-2 Blocking on Fetal Thymic Lobes[a]

| | % Positive[b] | |
|---|---|---|
| | Control | Anti-Il-2 |
| Exp. 1 | | |
| CD3−/CD4−/CD8− | 28.3 | 14.2 |
| IL-2R+/CD4−/CD8− | 15.8 | 0.3 |
| Exp. 2 | | |
| Total CD4−/CD8− | 25.4 | 12.9 |
| CD3+/CD4−/CD8− | 24.9 | 14.5 |
| CD3+/CD4−/CD8− | 2.3 | 2.7 |
| IL-2R+/CD4−/CD8− | 13.7 | 1.1 |
| CD5$^{dull}$/CD8− | 25.5 | 16.4 |
| Exp. 3 | | |
| CD3−/CD4−/CD8− | 20.7 | 13.1 |
| IL-2R+/CD4−/CD8− | 9.3 | 0.5 |
| CD5$^{dull}$/CD8− | 19.6 | 11.0 |

[a]Phenotypic analysis of intact day-14 fetal thymic lobes after anti-IL-2 (S4B6, Mossman et al., 1986) blocking in vitro following a 6-day incubation. The percent (%) positive indicates positive fluorescence over background, stained with an irrelevant antibody. Results shown are representative of five independent experiments.
[b]Day 14 FTOC, 6-day culture.

Taken together, our results show a marked increase in the percentage of CD3−/CD4−/CD8− thymocytes following IL-2 stimulation, or a decrease following IL-2 blocking, establishing IL-2 as a growth factor for these early immature precursor thymocytes. Thus, the same lymphokine that is responsible for the growth promotion of mature activated T cells also plays as a key role in the proliferation of fetal thymocytes.

### MATERIALS AND METHODS

### Mice

Timed pregnant C57BL/6 mice were obtained from The Jackson Laboratory (Bar Harbor, Maine). The day of observation of a vaginal plug was designated as day 0 of embryonic development. Pregnant female mice were killed by cervical dislocation and embryos dissected from the uterus. Thymus lobes were dissected from the embryos using a dissecting microscope and fine watchmaker's forceps.

### Hybridization Probes

All probes used for in situ hybridization were cDNA fragments cloned into the polylinker of the pGEM1 plasmid using strand procedures. The IL-2 probe was the 600 bp insert of pMIL-2-20 (Kashima et al., 1985) and was generously provided by T. Taniguchi. The murine IL-2R probe consisted of the full-length cDNA IL2R8 (Miller et al., 1985).

### In situ Hybridization

In situ hybridization was performed by a modification of the procedure of Harper et al. (1986), and as previously described (Pardoll et al., 1987). Briefly, fetal thymocytes from days 12–17 of gestation (timed pregnant C57BL/6 mice) were isolated, cytospun onto slides, and hybridized in situ with the [35-S]-labeled single-stranded RNA probe for IL-2 and IL-2R (Kashima et al., 1985; Miller et al., 1985). After a five-day exposure, slides were developed and counterstained with Giemsa. Grain count per cell was recorded for between 50 and 150 cells per slide. Nonspecific grain counts were determined by hybridizing under identical conditions with [35-S]-riboprobes synthesized from the opposite strand.

### Radiolabeled IL-2-Binding Assay

The radiolabled rIL-2-binding assays were performed as previously described (Teshigawara et al., 1987). Briefly, radiolabeled [125-I]-rIL2 was incubated together with cell suspensions ($10^6$ cells in 0.2 ml DMEM medium supplemented with 10% FCS), overlayed onto a 0.2-ml mixture of 80% silicone oil and 20% paraffin oil. After a 20-min incubation at 37°C, the tubes were centrifuged (8500 g for 90 s), the tips of the tubes containing the cell pellet were severed, and the cellbound and free radioactivity were determined by solid scintillation counting. The calculated values for the number of binding sites per cell were derived by Scatchard analysis of equilibrium binding data after substraction of nonspecific binding determined in the presence of 150-fold molar excess of unlabeled rIL-2.

### Fetal Thymus Organ Culture

Intact fetal thymus lobes were isolated from day 13–14 embryos and placed in organ culture as previously described (Mandell and Kennedy, 1978) or placed in culture in a 96-well microtiter plate, at one lobe per well. Lobes were incubated in Dulbecco's modified Eagle's medium supplemented with 10% FCS, 2 mM glutamine, 10 U/ml penicillin, 100 $\mu$g/ml streptomycin, 100 $\mu$g/ml gentamicin, 0.11 mg/ml sodium pyruvate, $5 \times 10^{-5}$ M 2-ME, and 10 mM Hepes. Cultures were incubated for 72 hr at 37°C in a humidified incubator containing 7.5% $CO_2$ in air.

### Proliferation Assays

Proliferation of fetal thymocytes in thymic organ culture was measured by incorporation of [3-H]-thymidine (1 $\mu$Ci/well) during a 6-hr pulse. Lobes were harvested and thoroughly washed with $ddH_2O$ and 95% ethanol, prior to processing for liquid scintillation counting. IL-2 responses were performed in triplicates; each point represents the arithmetic mean; the STD deviation was $<5\%$.

### Fluorescence Staining

Cell suspensions were prepared in HBSS (without phenol red) containing 1% BSA and 0.1% sodium azide (FACS buffer). Cells ($10^6$/100 $\mu$l buffer) were incubated on ice for 30 min with 10 $\mu$l of the appropriate antibody, and washed twice after each incubation. Control staining of cells, either with irrelevant antibody or with second antibody alone, was used to obtain background fluorescence values. The percent positive indicates positive fluorescence over background. The samples were analyzed on a FACS 440 (Becton and Dickinson, Mountain View, California) interfaced to a PDP 11/24 computer. Data were collected on 50,000 cells. Reagents used for direct staining were FITC- or biotin-conjugated anti-CD3 (Leo et al., 1987), -CD4 (Dialynas et al., 1983), -CD5 (Ledbetter and Herzenberg, 1979), -CD8 (Ledbetter and Herzenberg, 1979), -IL-2R (Ortega et al., 1984), and V-gamma-3 (Havran and Allison, 1988).

The relative DNA content was determined by fixing the cells (after staining for IL-2R) with ethanol followed by an incubation with RNase A and finally with propidium iodine; cells were washed and analyzed by flow cytometry, as previously reported (Ashwell et al., 1987).

### ACKNOWLEDGMENTS

We thank Drs. Jonathan D. Ashwell and Ron H. Schwartz for their valuable advice and discussions, and Fran N. Haussman and David A. Stephany of the NIAID Flow Cytometry Laboratory for their expert FCM analysis. This paper is in partial fulfillment of the Ph. D. degree requirement of Juan Carlos Zúñiga-Pflücker at George Washington University, Washington, D.C. 20052.

*(Received September 8, 1989)*

*(Accepted October 6, 1989)*

### REFERENCES

Ashwell J.D., Cunningham R.E., Noguchi P.D., and Hernandez D. (1987). Cell growth cycle block of T cell hybridomas upon activation with antigen. J. Exp. Med. **165:** 173–194.

Carding S.R., Jenkinson E.J., Kinston R., Hayday A.C., Bottomly K., and Owen J.J.T. (1989). Developmental control of lymphokine gene expression in fetal thymocytes during T-cell ontogeny. Proc. Natl. Acad. Sci. USA **86:** 3342–3345.

Ceredig R. (1986). Proliferation in vitro and interleukin production by 14-day fetal and adult Lyt2- and L3T4- mouse thymocytes. J. Immunol. **137:** 2260–2267.

Ceredig R. (1988). Differentiation potential of 14-day fetal mouse thymocytes in organ culture. Analysis of CD4−/CD8− defined single-positive and double-negative cells. J. Immunol. **141:** 355–362.

Ceredig R., Lowenthal J.W., Nabholz M. and MacDonald H.R. (1985). Expression of interleukin-2 receptors as a differentiation marker on intrathymic stem cells. Nature **314:** 98–100.

Ceredig R., Medveczky J., and Skulimowski A. (1989). Mouse fetal thymus lobes cultured in IL-2 generate CD3+, TCR-gamma-delta-expressing CD4−/CD8+ and CD4−/CD8− cells. J. Immunol. **142:** 3353–3360.

Denning S.M., Kurtzberg J., Le P.T., Tuck D.T., Singer K.H., and Haynes B.F. (1988). Human thymic epithelial cells directly induce activation of autologous immature thymocytes. Proc. Natl. Acad. Sci. USA **85:** 3125–3129.

Dialynas D.P., Quan Z.S., Wall K.A., Pierres A., Quintans J., Loken M.R., Pierres M., and Fitch F.W. (1983). Characterization of the murine T cell surface molecule, designated L3T4, identified by the monoclonal antibody GK1.5: similarity of L3T4 to the human Leu3/T4 molecule. J. Immunol. **131:** 2445–2451.

Fowlkes B.J., Edison L., Mathieson B.J., and Chused T.M. (1985). Early T lymphocytes: differentiation in vivo of adult intrathymic precursor cells. J. Exp. Med. **162:** 802–822.

Gillis S., and Smith K.A. (1977). Long term culture of tumor specific cytotoxic T cells. Nature **268:** 154–156.

Habu S., Okumura K., Diamantstein T., and Shevach E. (1985). Expression of interleukin 2 receptor on murine fetal thymocytes. Eur. J. Immunol. **15:** 456–460.

Hardt C., Diamantstein T., and Wagner H. (1985). Developmentally controlled expression of IL-2 receptors and of sensitivity to IL-2 in a subset of embryonic thymocytes. J. Immunol. **134:** 3891–3894.

Hardt C., Diamantstein T., and Wagner H. (1986). Detection of rearranged T cell receptor beta-chain gene and induction of cytolytic function in interleukin 2-responsive day 14–15 murine fetal thymocytes. Eur. J. Immunol. **16:** 1087–1092.

Harper M.E., Marselle L., Gallo R.C., and Wong-Staal F. (1986). Detection of lymphocytes expressing human T-lymphotropic virus type III in lymph nodes and peripheral blood from infected individuals by an in situ hybridization. Proc. Natl. Acad. Sci. USA **83:** 772–776.

Havran W.L., and Allison J.P. (1988). Developmentally ordered appearance of thymocytes expressing different T-cell antigen receptors. Nature **335:** 443–445.

Havran W.L., and Allison J.P. (1989). Expression and function of CD3 associated T cell receptors during intrathymic development. FASEB J. **3:** A365.

Jenkinson E.J., Kingston R., and Owen J.J.T. (1987). Importance of IL-2 receptors in intrathymic generation of cells expressing T-cell receptors. Nature **329:** 160–162.

Kashima N., Nishi-Takaoka C., Fujita T., Taki S., Yamada G., Hamuro J., and Taniguchi T. (1985). Unique structure of murine interleukin-2 as deduced from cloned cDNAs. Nature **313:** 402–404.

Ledbetter J.A., and Herzenberg L.A. (1979). Xenogenic monoclonal antibodies to mouse lymphoid differentiation antigens. Immunol. Rev. **47:** 63–90.

Leo O., Foo M., Sachs D.H., Samelson L.E., Bluestone J.A. (1987). Identification of a monoclonal antibody specific for a murine T3 polypeptide. Proc. Natl. Acad. Sci. USA **84:** 1374–1378.

Lowenthal J.W., Howe R.C., Ceredig R., and MacDonald H.R. (1986). Functional status of interleukin-2 receptors expressed by immature (Lyt 2− LeT3−) thymocytes. J. Immunol. **137:** 2579–2584.

Malek T.R., and Ashwell J.D. (1985). Interleukin 2 upregulates expression of its receptor on a T cell clone. J. Exp. Med. **161:** 1575–1580.

Mandell T.C., and Kennedy M.M. (1978). The differentiation of murine thymocytes in vivo and in vitro. Immunology. **53:** 317–331.

Miller J., Malek T.R., Leonard W.J., Greene W.C., Shevach E., and Germain R.N. (1985). Nucleotide sequence and expression of a mouse interleukin 2 receptor cDNA. J. Immunol. **134:** 4212–4218.

Mossman T.R., Cherwinski H., Bond M.W., Giedlin M.A., and Coffman R.L. (1986). Two types of murine helper T cell clone. I. Definition according to profiles of lymphokine activities and secreted proteins. J. Immunol. **136:** 2348–2357.

Ortega G., Robb R.J., Shevach E.M., and Malek T.R. (1984). The murine IL 2 receptor. I. Monoclonal antibodies that define distinct functional epitopes on activated T cells and react with activated B cells. J. Immunol. **133:** 1970–1975.

Pardoll D.M., Fowlkes B.J., Lechler R.I., Germain R.N., Schwartz R.H. (1987). Early genetic events in T cell development analyzed by in situ hybridization. J. Exp. Med. **165:** 1624–1638.

Papiernik M., Penit C., and Rouby S.E. (1987). Control of prothymocyte proliferation by thymic accessory cells. Eur. J. Immunol. **17:** 1303–1310.

Penit C., and Vasseur F. (1989). Cell proliferation and differentiation in the fetal and early postnatal mouse thymus. J. Immunol. **142:** 3369–3377.

Raulet D.H. (1985). Expression and function of interleukin-2 receptors on immature thymocytes. Nature **314:** 101–103.

Saragovi H., and Malek T. (1987). The murine interleukin 2 receptor. Irreversible cross-linking of radiolabeled interleukin 2 to high affinity interleukin 2 receptors reveals a noncovalently associated subunit. J. Immunol. **139:** 1918–1926.

Scollay R., Wilson A., D'Amico A., Kelly K., Egerton M., Pearse M., Wu L. and Shortman K. (1988). Developmental status and reconstitution potential of subpopulations on murine thymocytes. Immunol. Rev. **104:** 81–120.

Smith K.A. (1984). Interleukin 2. Annu. Rev. Immunol. **2:** 319–333.

Smith K.A. (1988). Interleukin-2: inception, impact, and implications. Science **240:** 1169–1176.

Tentori L., Pardoll D.M., Zúñiga-Pflücker J.C., Hu-Li J., Paul W.E. Bluestone J.A., and Kruisbeek A.M. (1988). Proliferation and production of IL-2 and B cell stimulatory factor 1/IL-4 in early fetal thymocytes by activation through Thy-1 and CD3. J. Immunol. **140:** 1089–1094.

Tentori L., Longo D.L., Zúñiga-Pflücker J.C., Wing C., and Kruisbeek A.M. (1988). Essential role of the interleukin 2-interleukin 2 receptor pathway in thymocyte maturation in vivo. J. Exp. Med. **168:** 1741–1747.

Teshigawara K., Wang H., Kato K., and Smith K.A. (1987). Interleukin 2 high-affinity receptor expression requires two distinct binding proteins. J. Exp. Med. **165:** 223–238.

Toribio M.L., Alonso J.M., Bárcena A., Gutiérrez J.C., de la Hera A., Marcos M.A.R., Márquez C., and Martínes-A C. (1988). Human T-cell precursors: involvement of the IL-2 pathway in the generation of mature T cells. Immunol. Rev. **104:** 55–79.

Toribio M.L., de la Hera A., Marcos M.A.R., Márquez C., and Martínez-A C., (1989). Activation of the interleukin 2 pathway precedes CD3-T cell receptor expression in thymic development. Differentiation growth requirements of early and mature intrathymic subpopulations. Eur. J. Immunol. **19:** 9–15.

von Boehmer H., Crisanti A., Kiesilow P., and Haas W. (1985). Absence of growth by most receptor-expressing fetal thymocytes in the presence of interleukin-2. Nature **314:** 539–540.

Watson J.D., Morrissey P.J., Namen A.E., Conlon P.J., and Widmer M.B. (1989). Effect of IL-7 on the growth of fetal thymocytes in culture. J. Immunol. **143:** 1215–1222.

